# Influence of bed occupancy and patient characteristics on bedspacing practices: a multicentre retrospective cohort study

**DOI:** 10.1136/bmjopen-2026-120864

**Published:** 2026-10-26

**Authors:** Wilhelm Linder, Ulrika Winblad, David Isaksson

**Affiliations:** 1 Department of Public Health and Caring Sciences, Uppsala Universitet, Uppsala, Sweden; 2 Health Services Research, Uppsala University, Uppsala, Sweden

**Keywords:** Bed Occupancy, Quality in health care, Hospitals, Safety

## Abstract

**Abstract:**

**Objectives:**

Hospitals cohort similar patients in departments. When departments are operating under high bed occupancy, patients may be relocated from the admitting department to a ward belonging to an off-service ward of a different clinical specialty. This process, often referred to as bedspacing, is not well understood and may have a negative impact on patient safety. As such, the objective of this study is to investigate the impact of patient characteristics and bed occupancy on bedspacing.

**Design:**

Retrospective cohort study.

**Setting:**

Nine hospitals in a Swedish healthcare region between 2015 and 2019.

**Participants:**

2 34 325 patients above the age of 18 who experienced unplanned hospital admission.

**Outcome measurements:**

Bedspacing operationalised as patients that were admitted to a cohorting department but physically located in an off-service ward.

**Results:**

The most important determinants of bedspacing were diseases of the genitourinary system (HR 1.23 (95% CI 1.16 to 1.31)) and musculoskeletal system (HR 1.37 (95% CI 1.27 to 1.47)). Patients with diseases of the circulatory system (HR 0.58 (95% CI 0.54 to 0.61)) and nervous system (HR 0.52 (95% CI 0.47 to 0.57)) were less likely to be bedspaced. Other patients characteristics such as age (HR 0.954 (95% CI 0.940 to 0.970 per SD increase), registered sex (HR 0.841 (95% CI 0.82 to 0.86) for men) and marital status (HR 1.194 (95% CI 1.16 to 1.23) for widows/widowers) were also found to influence bedspacing. Regarding bed occupancy, the HR of being bedspaced was 1.46 (95% CI 1.39 to 1.54) when comparing above 115% to less than 85% bed occupancy.

**Conclusions:**

Both clinical and non-clinical patient factors influenced bedspacing. Bedspacing was positively associated with bed occupancy with substantial variation across departments and operationalisations of bedspacing. Future research should differentiate between different types of bedspacing and bed occupancy to identify conditions under which bedspacing may be safe and equitable.

**STRENGTHS AND LIMITATIONS OF THIS STUDY**

- This study uses a comprehensive dataset from a regional healthcare system in Sweden including hospitals of different sizes and degree of clinical specialisation.
- The study incorporates two different operationalisations of bedspacing with implications for healthcare practice
- A limitation is that the routinely collected register-data does not contain information about disease severity, thus limiting disease severity adjustments.

## Introduction

 The number of available hospital beds per capita has been decreasing in most Organisation for Economic Co-operation and Development (OECD)-countries during the last decades.^1^ One major reason for the reduction in available hospital beds is the advancement of medical and surgical treatment to the extent where treatments that previously required inpatient care are now being performed in outpatient settings or in the patient’s own home.^2^ In addition, focus on productivity has shortened length of stay (LoS) in hospitals, with more patients being treated per hospital bed hour.^3^ However, there are counteracting trends that are increasing the need for hospital services. For instance, the majority of days spent in hospital occurs in the last year of life and increasingly older populations can therefore be expected to increase the demand for inpatient care. Furthermore, ageing populations have led to an increase in social and medical frailty causing bed blockages while patients are waiting for community health and social care services. In addition, medical advancements have increased the possibility of providing advanced treatments to more patients which in turn has caused hospital wards to become gradually more specialised.^4 5^ Consequently, many countries report strained healthcare systems where the demand for inpatient hospital services is higher than the available capacity and it thus appears as if bed capacity is declining faster than the savings from medical advancements have been able to achieve.^6^

### Management of hospital beds

When hospitals experience surges in patient inflow, the bed-occupancy of hospital wards increase. Previous studies have shown that high bed occupancy adversely affect hospital productivity and patient safety.^7–9^ For instance, high occupancy levels in hospital wards can result in access block, where patients have prolonged stays in emergency departments (EDs) while waiting for a hospital bed.^10^ Consequently, initiating time-critical treatments in hospital wards may be delayed.^11^

A common target in hospitals is to keep bed occupancy rates below 85%.^12^ For instance, the Swedish government agency National Board of Health and Welfare has published guidelines with a target of 85% bed occupancy for unplanned care and 95% for planned care.^13^ However, the ideal bed occupancy rate is contested, with some reports indicating that bed occupancy levels above 92.5% can be considered as ‘tipping-points’ where mortality starts to increase, in particular for the most severely ill patients.^14^ Other studies suggest that the ideal bed occupancy is dependent on the size of hospital and type of clinical specialty.^15–17^ Furthermore, as bed occupancy is an administrative measure of workload, it does not necessarily reflect the true workload as perceived by staff as it does not account for patient severity, nursing care need and patient turnover.^18^ Regardless of the ideal bed occupancy rate, managing overcrowding and high bed occupancy is essential in order to prevent adverse events for patients.^9 19^

Although the practice of bed management varies between hospitals and their departments, reducing bed occupancy levels in order to handle acute bed shortages is often achieved through a combination of several different measures. First of all, the number of available staffed hospital beds can be increased by calling in more staff or by postponing planned care. Another option is to discharge patients earlier than planned; however, since premature hospital discharge is a known cause of readmission, this option may only provide temporary relief for hospital staff.^20^ If the above options are not sufficient, it may become necessary to board patients in EDs and/or exceed available capacity, with negative consequences for both staff and patients.^10 21^

### Bedspacing

Although it is possible to overcrowd hospital departments with bed occupancies above 100%, high bed occupancy will eventually cause turn-away, that is, where patients are diverted to other hospitals or boarded in EDs while waiting for admission to a hospital ward.^15^ Another form of turn-away is when patients admitted to a particular clinic department (cohorted based on diagnosis) are transferred to off-service wards belonging to clinics with different clinical specialties. Depending on the country, this process is often referred to as boarding, off-service placement or bedspacing. In general, the medical responsibility for a bedspaced patient remains with the admitting department, while medication administration, monitoring and nursing care are delivered by staff on the off-service ward. Bedspacing can occur early in a hospital spell, for example, when a patient is still in the ED and the most appropriate admitting department lacks available capacity. Patients can also be bedspaced towards the end of the hospital spell where the patient has been stabilised after diagnostics and initiation of treatment. In practice, the latter type of bedspacing is often considered to be less of a concern compared with being bedspaced immediately after visiting the ED.

As with early discharge, bedspacing to off-service wards can be used as a tool to decrease bed occupancy at a particular department and, in theory, protect the most severely ill patients in the admitting department.^22^ However, there may be a risk-risk trade-off as bedspacing has been associated with negative effects on both patient safety and healthcare quality.^23^ The concern is that the discrepancy between the patient’s clinical needs and the expertise available among the clinical and nursing staff in the off-service ward may result in, for example, medication errors, inadequate patient monitoring, procedural errors, postoperative complications and delays in treatment initiation.^23–26^ Indeed, a systematic review by La Regina *et al*, indicates that bedspaced patients have a higher risk of readmissions, in-hospital mortality and longer LoS.^23 27^ However, other studies report no harm and, in some instances, even protective effects from bedspacing, raising questions regarding whether geographical cohorting is necessary or not for certain groups of patients.^23 28 29^

These inconsistencies are likely related to differences between studies regarding the types of patients and departments that have been studied.^30^ For instance, the majority of studies investigating the effects of bedspacing include patients that are bedspaced from^27 28 31 32^ general internal medicine departments with few studies including patients bedspaced from surgical or other types of departments.^22 33^

Furthermore, it is often assumed that patients are classified as eligible for bedspacing primarily based on disease severity and nursing care needs, with bedspacing mainly reserved for relatively medically ‘fit’ patients.^31^ However, the assumption that bedspaced patients are more ‘medically fit’ can be questioned. In Sweden, there are relatively few hospital beds per capita with hospital treatments being mainly reserved for patients with acute and severe illness.^1^ As such, there may be few ‘medically fit’ patients that can be placed in off-service wards and the decision to bedspace may instead be based on whether the necessary treatment can be performed elsewhere or not. Furthermore, previous studies indicate that patients with lower socioeconomic status are more likely to experience adverse events during hospital care.^34 35^ However, there is a knowledge gap regarding whether socioeconomic status influences bedspacing and whether the increased risk of adverse events is mediated by a higher frequency of intrahospital transfers.^32^

Lastly, there is a lack of consensus regarding the operationalisation of bedspacing. In the scientific literature, a broad operationalisation is commonly used where patients transferred to a department of a different (sub)specialty are considered bedspaced. In practice, there may be an assumption that there is sufficient inter-operability within medical or surgical disciplines and that such transfers are not seen as bedspacing.^36^

In theory, the interoperability of wards within medical (or surgical) disciplines might allow for safer bedspacing compared with bedspacing *between* medical and surgical disciplines. However, if the perceived safety of bedspacing *within* disciplines leads to a drift in which types of patients that are considered eligible for bedspacing (ie, more severely ill patients), there may still be negative impacts on patient safety. In summary, little is known about different types of bedspacing and if the type of bedspacing has different impacts on patient safety.

### Study rationale and research objectives

The uncertainty regarding the effects of bedspacing makes it difficult to assess the risk-risk trade-off between bedspacing patients to off-service wards instead of increasing bed occupancy at the admitting department. As such, more knowledge is needed about which factors determine bedspacing, both at the patient and hospital level. Addressing this knowledge gap is important for both researchers examining the effects of bedspacing on patient outcomes as well as for healthcare practitioners working with hospital bed management.^27 28^

The primary objective of the study is therefore to investigate the impact of patient characteristics and bed occupancy on bedspacing. The secondary objective is to examine these relationships using a stricter operationalisation of bedspacing.

## Methods

### Study design

The study is designed as a multicentre retrospective cohort study. Strengthening the Reporting of Observational Studies in Epidemiology (STROBE) reporting guidance has been followed in the reporting of this study.

### Setting

The study was performed in a Swedish healthcare region with a catchment area of more than 1 million people. The healthcare region has nine hospitals including a university hospital with approximately 1100 staffed beds that provided almost 50% of the hospital spells in the dataset (see table 1). The Swedish healthcare system is characterised by relatively few staffed hospital beds per capita. Instead, there has been a shift towards outpatient care with inpatient care mainly reserved for the most severely ill patients. In practice, hospital departments are often divided into inpatient care (one or more wards together representing the departments bed pool) and outpatient care clinics that will handle non-acute diagnostics and follow-up care after hospital discharge. Furthermore, the decision to admit and bedspace a patient is taken by attending physicians at the ED and relevant clinical department. After that, bed managers are tasked with finding a suitable bed taking into account both medical as well as nursing care needs.

**Table 1:** Characteristics of included hospitals

Hospital	Category	Number of departments	Patient days in study	Bed occupancy (mean, %)	Bedspacing events
A	University hospital	35	1 413 120	93.3	23 092
B	Acute hospital	11	515 684	103.6	6483
C	Acute hospital	14	409 658	91.3	2171
D	District hospital	4	164 134	97.0	3171
E	District hospital	10	113 313	84.0	278
F	District hospital	2	98 911	97.3	264
G	District hospital	2	134 668	85.4	47
H	District hospital	2	104 403	89.5	47
I	District hospital	2	50 786	75.7	25

### Study population

The study sample consists of patients who were admitted for somatic care in any of the included hospitals between 1 January 2015 and 29 February 2020. Hospital spells were excluded if they were planned/elective, if the patient was below 18 years of age or if treatment was provided in palliative, maternity or paediatric wards. The study period was restricted to 29 February 2020 as the COVID-19 pandemic caused organisational changes within the studied hospitals that were not always registered in the administrative systems.

### Data collection

Clinical patient data were collected from electronic health records kept by the Swedish healthcare region. Data was collected in the form of hospital care episodes which consisted of an admission to a hospital department up until discharge (or death) from the same department. Hospital care episodes that followed each other without the patient leaving the hospital were linked to generate continuous hospital spells.

Data regarding admitting and off-service departments, wards and number of staffed beds were collected from central administrative systems in the healthcare region. Data regarding socioeconomic status were collected from the Swedish total population registry maintained by the government agency Statistics Sweden.^37^ Data linkage between data sources was performed by Statistics Sweden using personal identification numbers.^38^

### Variables

#### Bed occupancy

Bed occupancy was calculated at the department level and defined as the sum of staffed beds in all wards belonging to a department in relation to the number of patients physically present in these wards (including planned visits). The assessment of the number of staffed beds available varied across departments but was generally determined by the department’s chief physician based on an assessment of the number of patients that the staff in each ward could reasonably be expected to care for.

Bed occupancy was measured at 11:00 which was when bed occupancy peaked during the day, thus representing the busiest time of the day where there is a high rate of admissions and discharges simultaneously.^39 40^ Patients treated in intensive care units were excluded from bed occupancy calculations. For a visual representation of the 24-hour cycle of bed occupancy, see online supplemental figure 1.

Bed occupancy was further categorised as below 85%, 85–100%, 100–115% and above 115% based on recommended levels of bed occupancy in Sweden and available bed occupancy data (see online supplemental figures 2 and 3).

#### Patient characteristics

Patient characteristics included both clinical and non-clinical factors. The clinical factors consisted of age (standardised), registered sex (female/male), diagnosis and multimorbidity (weighted Charlson Comorbidity Index (CCI)).^41^ The primary diagnosis of a hospital spell was classified using the International Statistical Classification of Diseases and Related Health Problems 10th Revision (ICD-10) by converting the dominant ICD sub-category (most common primary diagnosis) during the hospital spell to its corresponding ICD-chapter.

Non-clinical patient characteristics were collected from Statistics Sweden. Education was operationalised as the highest level of education a person started and was categorised as completed primary school, secondary school (gymnasium) or tertiary education (university studies or vocational training). Marital status was categorised according to if the patient at the time of the hospital visit was married, divorced, unmarried or a widow(-er). Finally, the region of birth was categorised based on whether the patient was born in Sweden, Europe (European Union, Switzerland, Norway or Iceland) or outside of Europe.^42^

#### Bedspacing

Bedspacing was operationalised as patients being admitted to a cohorting department but physically located in a ward belonging to an off-service department. Off-service departments were defined as departments of another (sub)discipline than the admitting department, including both surgical and medical specialties (see online supplemental table 1). Furthermore, patients were only considered to be bedspaced if the off-service department was of a similar level of care. As such, patients transferred to intensive care or intermediate care units were not considered as bedspaced. Exceptions from the general rules above included transfers between highly specialised departments (ie, neurosurgery and thoracic surgery) to neurology or cardiology due to the mixed nature of these departments and wards.

For the secondary research objective, bedspacing followed the operationalisation used by the healthcare region where patients are only considered bedspaced if there is insufficient interoperability between departments. As a general rule, patients were not considered bedspaced if the off-service ward belongs to a department with the same primary treatment modality (ie, surgical or medical) see online supplemental table 1).

### Statistical analysis

#### Descriptive statistics

Patient characteristics were presented using descriptive statistics. In addition, descriptive statistics were used to calculate the number of ED visits during the last 12 months, complications of care during spells (ICD-codes T80–T88) and total length of hospital stay for cohorted and bedspaced patients. Disease-related group (DRG) scores were provided by the healthcare region.

#### Inferential statistics

The initial data consisted of hospital spells ranging from the first day of admission to a hospital department up until an event occurred (death, bedspacing or discharge from the hospital). For the analysis, we assumed that the immediate effect of bed occupancy (ie, same day) was more important for bedspacing than the cumulative exposure throughout a hospital spell. Hospital spells were therefore expanded into daily patient observations (analytical unit), thus allowing bed-occupancy at the admitting department to be treated as a time-varying exposure. For days where patients changed departments within the same hospital spell, the department where the patient spent the majority of the day was used as the daily observation. Patient-days where there was no available data regarding the number of staffed beds at the admitting department were excluded from the analysis. The effect of bed occupancy on bedspacing was then modelled using discrete time-survival models with time-at risk included as the number of days since the first day of hospital admission.^14^

In total, four models were used to explore the relationships between the chosen variables and bedspacing. Model 1 (M1) was a model that investigated the impact of bed occupancy on bedspacing while including the day of the hospital visit for patients. The relationship between bed occupancy and bedspacing in M1 was also modelled using natural splines to allow for non-linear effects (generalised linear model) on a continuous scale of bed occupancy. To aid with interpretation, predicted probabilities were calculated across values of bed occupancy.

Model 2 (M2) included clinical variables such as age, registered sex and ICD-chapter of main diagnosis. In the third model (M3), an additional set of variables was included in order to explore the potential impact of socioeconomic status. Finally, model 4 (M4) expanded on M3 by including random effects of departments.

The reasoning behind including patient characteristics was twofold. First, these covariates were included in order to investigate the effects of patient characteristics on bedspacing. Second, due to the potential of selection bias, adjusting for clinical characteristics of patients is likely necessary in order to generate unbiased estimates of the impact of bed occupancy on bedspacing. The problem of selection bias is related to changes in admission processes with previous studies indicating that the admission rate of patients from the ED decreases at higher bed occupancies.^43 44^ This shift in admission practices could therefore introduce selection bias if there is a shift towards primarily admitting the most severely ill patients, that is, patients that are less likely to be bedspaced. As the diagnosis of the patient is assumed to be a strong predictor of bedspacing, the potential differences in disease distributions (ICD-codes) at varying levels of bed occupancy will likely influence the results. In this case, ICD-codes are not confounders in a causal sense as the type of disease is not a direct cause of bed occupancy. However, due to the changes in admission processes at high bed occupancy, ICD-codes will be associated with bed occupancy and therefore behave, in practice, like confounders in the statistical models.

In the fourth and final model (M4) we assumed that departments vary in (1) baseline bed occupancy and (2) which threshold of bed occupancy bedspacing becomes the preferred option compared with increasing bed occupancy. Due to the specialisation of departments, fixed effect modelling at the department level was deemed less feasible as there would be few reference patients (ICD-chapter) available for comparisons within most departments. Instead, M4 used a random intercept model accounting for both variation between and within departments. Finally, an exploratory analysis was performed using the M1 model and natural splines with the dataset split between the large university hospital and the smaller hospitals.

All analyses were performed in R V.4.5.3 using the packages glm, glmmTMB, splines and marginal effects.

### Patient and public involvement

None.

## Results

After applying exclusion criteria to the dataset (see online supplemental table 2), the final study sample consisted of 3 040 255 patient days representing 480 245 hospital spells from 234 325 patients and 82 departments (see tables 1 and 2). Bedspacing occurred in 35 578 hospital spells with a majority (74%) occurring on the first day of hospital admissions (see table 2). Among the bedspaced patients, a majority (74%) were bedspaced on the first day of admission. Bedspaced patients were more likely to be female, had lower CCI scores and more ED-visits within the last 12 months. Bedspaced patients also differed from cohorted patients in the frequency of diagnoses measured as ICD-chapters (see online supplemental table 3). Patients with diseases of the circulatory system (I00–I99) or the nervous system (G00–G99) were less common in the bedspaced group compared with the cohorted group. On the opposite, patients with diseases of the digestive system (K00–K93), consequences of external causes (S00–T98) and disease of the genitourinary system (N00–N99) were more common in the bedspaced group.

**Table 2:** Characteristics of patients in the included hospital spells

Characteristic	Overall spells, N=480 245*	Cohorted spells, N=444 667*	Spells with bedspacing, N=35 578*	P value†
Age (mean, SD)	68 (19)	68 (19)	68 (21)	<0.001
Female (%)	243 770 (51)	224 191 (51)	19 579 (55)	<0.001
CCI (mean, SD)	1.21 (1.66)	1.22 (1.65)	1.19 (1.72)	<0.001
DRG (mean, SD)	0.91 (0.94)	0.91 (0.94)	0.98 (0.90)	<0.001
Days to event‡ (mean, SD)	6 (7)	7 (8)	2 (4)	<0.001
Length of stay§ (mean, SD)	8 (8)	8 (9)	8 (7)	<0.001
ED visits last 12 months (mean, SD)	1.44 (2.98)	1.44 (2.98)	1.52 (3.06)	<0.001
Care in ICU (%)	30 463 (6.3)	29 878 (6.7)	585 (1.6)	<0.001
Complication of care during hospital visit (%)	12 829 (2.7)	11 560 (2.6)	1269 (3.6)	<0.001
Bedspaced at hospital admission	26 310 (5.5)	0 (0)	26 310 (74)	<0.001
Bedspaced at hospital discharge¶	5753 (1.2)	0 (0)	5753 (16)	<0.001

* Number of hospital spells.

† Wilcoxon rank-sum test; Pearson’s χ2 test.

‡ Day to event was calculated as the number of days from admission to event (death, discharge, bedspaced).

§ Length of stay was calculated as the number of days from first day of hospital spell up until being discharged from the hospital.

¶ Bedspaced at hospital discharge indicates whether the patient was bedspaced in the final day of the hospital spell.

CCI, Charlson Comorbidity Index; DRG, disease-related groups; ED, emergency department; ICU, intensive care unit.

Furthermore, complications of care were higher in bedspaced patients which was also reflected in the higher DRG scores. On the contrary, treatment in ICUs was lower among bedspaced patients, reflecting that the most severely ill patients are rarely eligible for bedspacing other than perhaps at the end of a hospital spell after the patient has been stabilised.

In the 15 most common pairs of departments involved in bedspacing, general internal medicine, surgery and orthopaedic surgery were the most common cohorting departments (see figure 1). Off-service wards were more diverse, including infectious disease and plastic surgery wards. Regarding bed occupancy, the majority of daily observations at the respective departments were above 85% (see online supplemental figure 1).

**Figure 1:**
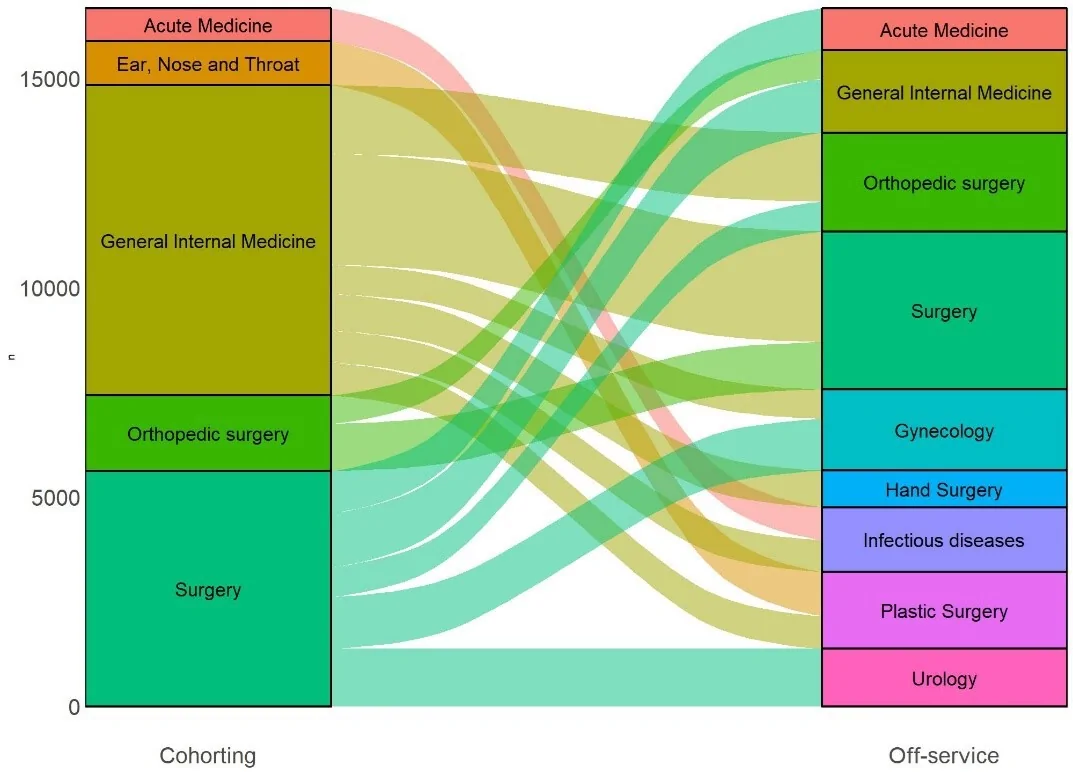
Pairs of cohorting and off-service wards involved in bedspacing. Alluvial plot showing the 15 most common pairs of clinics that are involved in bedspacing in the primary analysis. The left columns represent the cohorting clinic that are medically responsible for the patient whereas the right column represents the off-service clinic where the patient is physically present. The thickness of the ribbons represents the relative number of patients who were bedspaced in the particular pair of departments.

Regarding clinical patient characteristics, the HRs of being bedspaced varied significantly for patients with different diseases, broadly reflecting the disease severity and/or complexity of treatment (see table 3 and online supplemental table 4). Patients with diseases of the circulatory or nervous system had lower HRs of being bedspaced compared with patients with diseases of the genitourinary or musculoskeletal system. Furthermore, patients with higher CCI-scores had lower hazard of being bedspaced, however the statistically significant effect disappeared in M4.

**Table 3:** Effects of patient characteristics and bed occupancy on bedspacing

Variable	Model 1	Model 2	Model 3	Model 4
Bed occupancy (Ref: <85%)				
85–100%	1.939 (1.87 to 2.01)	1.811 (1.75 to 1.87)	1.807 (1.75 to 1.87)	1.036 (1 to 1.07)
100–115%	2.422 (2.34 to 2.51)	2.165 (2.09 to 2.25)	2.163 (2.08 to 2.24)	1.187 (1.14 to 1.24)
>115%	3.967 (3.79 to 4.15)	3.357 (3.21 to 3.52)	3.356 (3.2 to 3.51)	1.46 (1.39 to 1.54)
Day of visit	0.678 (0.67 to 0.68)	0.682 (0.68 to 0.69)	0.682 (0.68 to 0.69)	0.700 (0.7 to 0.7)
Registered sex (Ref: Female)				
Male		0.828 (0.81 to 0.85)	0.852 (0.83 to 0.87)	0.841 (0.82 to 0.86)
Age (scaled)		0.927 (0.92 to 0.94)	0.927 (0.92 to 0.94)	0.954 (0.94 to 0.97)
CCI (weighted)		0.987 (0.98 to 0.99)	0.988 (0.98 to 1)	0.993 (0.99 to 1)
Marital status (Ref: Married)				
Divorced			1.059 (1.03 to 1.09)	1.024 (0.99 to 1.05)
Unmarried			1.15 (1.11 to 1.19)	1.089 (1.05 to 1.12)
Widow(er)			1.213 (1.18 to 1.25)	1.194 (1.16 to 1.23)
Region of birth (Ref: Sweden)				
Europe			1.101 (1.07 to 1.14)	1.037 (1.00 to 1.07)
Outside of Europe			1.122 (1.07 to 1.18)	1.041 (0.99 to 1.09)
Number of departments				74*
AIC	347 459.4	342 128.9	341 905	310 766.2

M1 is a model of the association between bed occupancy and bedspacing. M2 includes adjustment for clinical patient characteristics with M3 also adjusting for variables related to socioeconomic status. M4 is a mixed effects model and includes a random intercept for department. Random effects are reported as SD. The effects are reported as HRs with 95% CIs in parenthesis.

*Note:* ICD-codes were not available in 0.2% of the observed patient days. These observations were excluded from models M2 to M4.

* Some departments placed their patients on shared wards. Out of the 82 departments included, there were 74 distinct bed pools. Patients that were transferred between these departments while remaining in the same bed pool were not considered to be bedspaced.

AIC, Akaike Information Criterion; CCI, Charlson Comorbidity Index; ICD, International Classification of Diseases.

Other than ICD-chapters, the strongest determinant of bedspacing was registered sex, with men having lower hazard of bedspacing compared with women (HR 0.841 (95% CI 0.82 to 0.86)). Additionally, the hazard of being bedspaced decreased by increasing age (HR=0.954 (95% CI 0.940 to 0.970) per SD increase). For the factors related to socioeconomic status, the hazard increased for widow(er)s (HR 1.194 (95% CI 1.16 to 1.23)) and patients born outside of Sweden whereas there was no effect from the level of education or region of birth in M4.

As seen in figure 2, the probability of bedspacing increased with higher levels of bed occupancy. The probability of bedspacing was relatively low when bed-occupancy was below 100% and then increased non-linearly at higher bed occupancies. The results also indicate that there was high variability between departments as the HR of being bedspaced at a bed occupancy above 115% decreased from 3.356 (95% CI 3.2 to 3.51) in M3 to 1.46 (95% CI 1.39 to 1.54) in M4. Bedspacing behaviour differed between smaller and larger hospitals, where larger hospitals had a higher probability of bedspacing at lower bed occupancies compared with smaller hospitals (see online supplemental figure 4 and online supplemental table 5).

**Figure 2:**
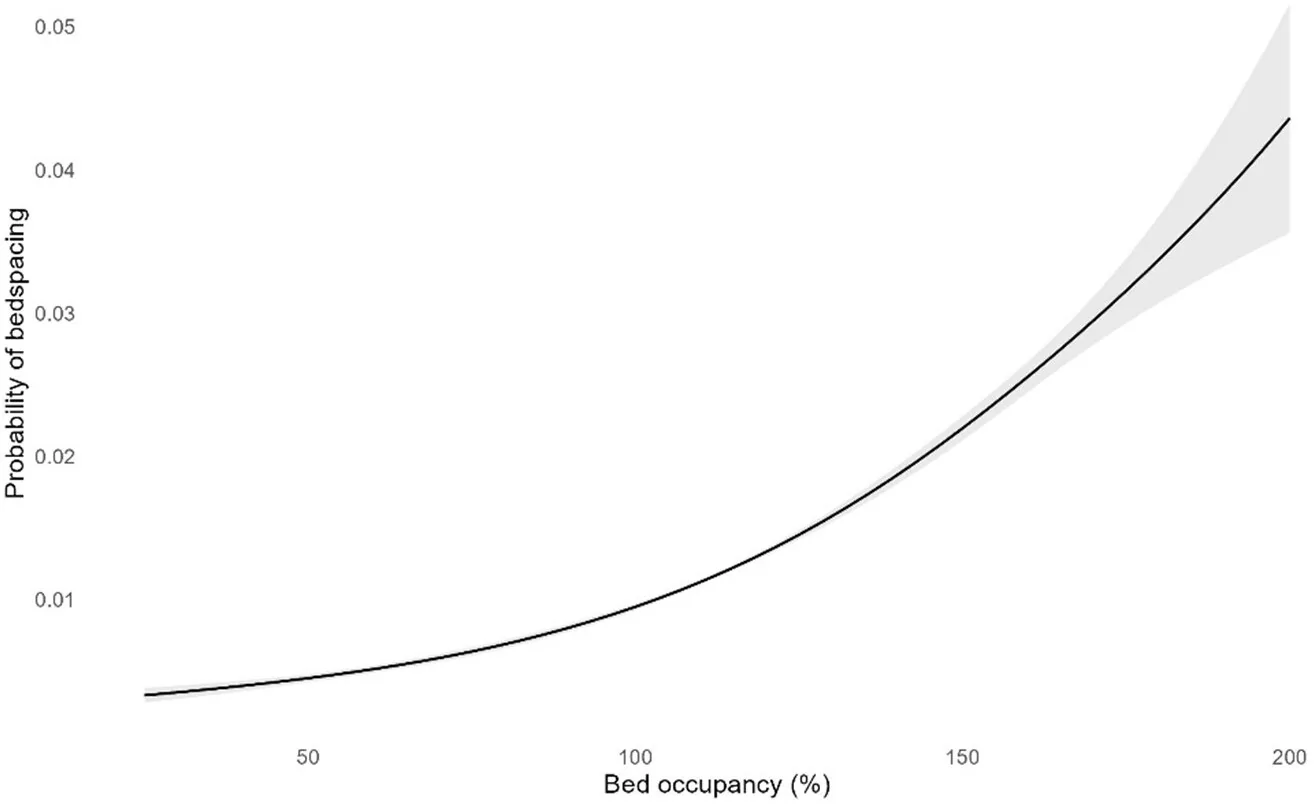
Predicted probabilities of bedspacing. Predicted probabilities of bedspacing in relation to bed occupancy based on a univariate generalised linear model using natural splines. Grey ribbon shows 95% CI.

Finally, the clinical characteristics of bedspaced patients were similar under the stricter operationalisation of bedspacing, with no major differences in the composition of diagnoses or the hazard of being bedspaced (see online supplemental tables 6-7 and online supplemental figure 5). However, the HR of bedspacing was consistently higher in relation to bed occupancy compared with the broader operationalisation of bedspacing.

## Discussion

The first primary research objective was to investigate the impact of patient characteristics and bed occupancy on bedspacing. The findings suggest that the composition of bedspaced patients was similar to patterns reported in previous literature, with diseases of the digestive and genitourinary systems being overrepresented in bedspaced patients. However, for some ICD-chapters there were reversals in the effects between M3 and M4 (eg, from HR 1.44 to 0.77 for patients with diseases of the digestive system) indicating substantial variation in bedspacing practices between departments.

Similarly, the results indicate that older patients were less likely to be bedspaced and that intensive care was less common among bedspaced patients. These findings are in line with the assumption that patients requiring less complex care are more likely candidates of being bedspaced.

The finding from our study that women were over-represented in bedspaced patients has only been reported in one of the few studies including patients bedspaced from surgical departments.^32^ This suggests that the focus on bedspacing from medical disciplines in previous studies might have overlooked a potential patient safety issue for women treated at surgical departments. However, this finding should be interpreted with caution as there might be residual confounding due to unmeasured disease severity or acuteness of disease.

Furthermore, previous studies about the effects of bedspacing have mainly included clinical covariates with only few studies accounting for socioeconomic factors.^31 32^ The results from our study showed that non-clinical factors such as marital status may influence the decision to bedspace patients. In addition, complications of care were higher in the bedspaced group, possibly explaining the mechanism behind increased length of hospital stay and readmissions among bedspaced patients reported in previous studies. However, these results should be interpreted with caution as it may be highly dependent on reporting procedures of different departments (possible reporting bias in bedspaced patients) and differences in how socioeconomic factors are quantified in different countries. Nevertheless, these findings warrant further investigation as it may have implications for equity in healthcare as well as methodological implications for future studies about bedspacing.

Another determinant of bedspacing was bed occupancy with HRs for bedspacing reaching, for example, 3.36 (95% CI 3.2 to 3.51) in M3 when comparing >115% to <85% bed occupancy. However, the results from the model that included random intercepts for departments (M4) showed substantially lower associations, with HR 1.46 (95% CI 1.39 to 1.54) in the total sample and 1.834 (95% CI 1.7 to 1.98) in the stricter operationalisation of bedspacing at >115% bed occupancy. This finding indicates that the effect of bed occupancy on bedspacing was to a large extent driven by baseline differences between departments. This likely reflects both differences in disease severity of patients between departments and possibly, although speculative, different preferences regarding at which threshold of bed occupancy bedspacing becomes the preferred option over increasing bed occupancy.

However, the interpretation of the relationship between bed occupancy and bedspacing is difficult. As seen in figure 2, bedspacing starts to increase before 100% bed occupancy indicating that clinicians aim to avoid 100% bed occupancy and retain some available capacity in order to admit new patients and/or to protect the most severely ill patients (see figure 2).^4 14^ The probability of bedspacing increases substantially at bed occupancies above 100%, indicating that bedspacing mainly becomes an option after departments have reached full capacity. This may be due to the high overall bed occupancy seen in online supplemental figure 1, effectively limiting the possibility to bedspace patients. This is further confirmed by the finding that bedspacing only occurred in 7.4% of hospital spells which is considerably lower compared with other studies.^22 28 31^ Furthermore, departments in the large hospitals appear to behave differently from departments in smaller hospitals. As seen in online supplemental figure 4 and online supplemental table 5, bedspacing starts to increase at lower bed occupancy in the large hospitals. Although speculative, it is possible that bedspacing is relatively difficult in smaller hospitals as there are fewer off-service wards with similar clinical expertise. As such, bedspacing may have to occur between different disciplines (eg, from medical to surgical or vice versa) and may be seen as high-risk bedspacing which should be avoided as long as possible.

In summary, the findings are important for the future direction of research on bedspacing and its potential impact on patient safety. It has previously been suggested that future studies of bedspacing should focus on investigating when cohorting is necessary and use non-inferiority designs.^29^ Given the results of the present study, we would like to add to this by proposing that future studies should use causal inference frameworks and carefully consider the most clinically relevant counterfactual, that is, comparing bedspaced patients to patients treated in departments that have exceeded full capacity.^27 28^ The knowledge gained from such studies would be of great importance for clinicians dealing with hospital bed shortages.

In addition, the HRs of being bedspaced at above 100% and 115% bed occupancy were higher in the stricter operationalisation of bedspacing. This finding suggests that bedspacing *between* medical and surgical disciplines are perceived as more harmful and performed out of necessity (high bed occupancy) whereas potentially safer bedspacing *within* medical or surgical disciplines may be performed for other reasons (eg, in anticipation of high bed occupancy levels and/or appropriate treatment of comorbidities). As hospital capacity challenges are likely to remain a problem, we believe that it is important to continue the work by Zannella *et al* and analyse distinct types of bedspacing as this might enable futures to elucidate in which circumstances, if any, bedspacing is possible without compromising patient safety.^28^

### Strengths and limitations

A strength of the study is the extensive dataset, including patients from an entire healthcare region within both medical and surgical departments. This, in combination with the different operationalisations of bedspacing, allows for a comprehensive picture of bedspacing not previously described. However, despite including several important variables for patient characteristics, the data did not allow precise assessments of disease severity or acuteness. As disease severity is most likely a mediator in the relationship between patient characteristics and bedspacing, it is possible that this data limitation can bias the results. Furthermore, the study calculated bed occupancy using the number of patients in relation to the number of staffed beds, thus providing an accurate measurement of bed occupancy. However, the reliability of this measurement is uncertain as the assessment of the number of staffed beds needed may differ between departments. In addition, bed occupancy does not always reflect nurse workload and the results should therefore be interpreted with caution. However, due to the Swedish healthcare systems shift towards outpatient care and few staffed hospital beds, hospital care is often reserved for the most ill patients with high nursing care needs. As such, the bed occupancy measurement used in this study is likely highly correlated with nurse workload.

On a similar note, although this study applied multilevel modelling using both data at the patient and department level there may still be unmeasured factors at the department level that could explain why certain patients are bedspaced more often than others. For instance, departments that are often operating at high bed occupancies may have collaborations, and therefore high-interoperability, with particular off-service wards with care for bedspaced patients being more or less equivalent to care in the cohorting department. As such, variation in local practices between departments in different types and sizes of hospitals might limit the generalisability of the results.

## Conclusions

Clinical patient characteristics such as disease type, age and registered sex are important determinators of bedspacing. In addition, non-clinical factors such as marital status also influenced bedspacing with possible implications for healthcare equity. The HR of being bedspaced increased with higher levels of bed occupancy but demonstrated high variability between departments and between different operationalisations of bedspacing. Future studies of bedspacing should carefully consider different types of bedspacing and bed occupancy in order to elucidate in which instances bedspacing might be performed safely.

## Supplementary material

10.1136/bmjopen-2026-120864 online supplemental file 1

## Data Availability

Data may be obtained from a third party and are not publicly available.
