# Supplementary material for: Influence of bed occupancy and patient characteristics on bedspacing practices: a multicentre retrospective cohort study
Source: BMJ Open. 2026 Oct 26;16(10):e120864. doi: 10.1136/bmjopen-2026-120864 (PMC13711533; doi:10.1136/bmjopen-2026-120864)
Supplement: Supplementary file 1 [file bmjopen-2026-120864.reviewer_comments.pdf]

PEER REVIEW HISTORY

BMJ Open publishes all reviews undertaken for accepted manuscripts. Reviewers are asked to complete a checklist review form and are provided with free text boxes to elaborate on their assessment. These free text comments are reproduced below.

ARTICLE DETAILS

Title (Provisional)

Influence of bed occupancy and patient characteristics on bedspacing practices: a multicentre retrospective cohort study

Authors

Linder, Wilhelm; Winblad, Ulrika; Isaksson, David

VERSION 1 - REVIEW

**Reviewer**	**1**
**Name**	**Newnham, Evan**
**Affiliation**	**Eastern Health**
**Date**	**08-Jun-2026**
**COI**	**None**

This is interesting subject matter not previously explored in this way.

Introduction: Is there evidence that the maturation of hospital in the home models of care has also reduced demand on inpatient capacity (may strengthen the introduction).

The term 'bedspacing' is not in common usage in the antipodes - an early reference to the terminology would help the non-European reader. The introduction (particularly the 'bedspacing' section goes into significant detail regarding patient outcomes related to bedspacing and could be substantially shortened.

Note the inconsistency in dates in the 'study population section' - please address.

The term 'sex' is used a few times and could be replaced with 'gender'.

A substantial components of the results discuss the clinical outcomes of the patient cohorts - noting the primary purpose of the paper is concentrated on demographic differences, the results section could also be shortened.

As a general comment, the paper could be shortened to focus more on the stated main objectives.

**Reviewer**	**2**

**Name**	**Jones, Rodney**
**Affiliation**	**Healthcare Analysis & Forecasting**
**Date**	**15-Jul-2026**
**COI**	**None**

This is a valuable study which identifies the conditions, ages and socioeconomic factors associated with patients placed outside their most suitable specialty.

May I suggest some improvements to enhance the study:

* I had not come across the term bed spacing before. In the USA this is called off-service placement and in the UK called bed boarding or outliers It may be useful to see if any other terms are used and inform the readers accordingly. Perhaps point out that the terminology varies around the world. In the UK the term boarding can be used in both an ED and inpatient context. Also, in the UK the term outlyer is also used in an inpatient context. You may have missed relevant UK studies due to terminology.

* In the introduction you need to mention that average bed occupancy has also been increasing over time indicating that bed supply is declining faster than than the savings from technological change.

* You probably need to mention the nearness to death or time to death effect where 55% of a persons lifetime bed occupancy occurs in the last year of life irrespective of the age at death. In this case the trend in the absolute number of deaths becomes a predictive variable in bed demand.

* The issue of hospital bed occupancy needs further elaboration. One study titled 'Hospital bed occupancy demystified' may give you some leads. More recent studies on the topic are available and the term 'turn-away' may be worth pursuing.

* Turn-away and bed occupancy are interlinked and depend on the size of the hospital, hence, you need to give some detail regarding the size of the hospitals in the study and check if the off-service placement is affected by hospital size.

* Indeed the 85% occupancy rule has no proof and has been shown to be entirely inappropriate for small units. In the literature 85% occupancy is incorrectly used as a proxy for staff busyness. Busyness generates a range of adverse outcomes but does not generate turn-away per se.

* Other than the above comments the other parts of the introduction are excellent and very comprehensive.

Regarding beds in Sweden it has been confirmed that Sweden has the lowest level of available beds per death in the developed world, as per the nearness to death argument above. In this study deaths per 1000 population was used as a measure of population age profile from a nearness to death perspective, i.e. age per se is an unreliable indicator.

* Socioeconomic status needs to be qualified as different measures can be used in different countries.

** **

Further comments:

* The most important issue lies around the theoretical basis for this behaviour. The figure of 85% has no theoretical basis and absolutely does not apply to small departments such as maternity, paediatrics or critical care. Strictly speaking it only approximately applies to very large hospitals but not to the bed pools within these hospitals.

* In the literature % occupancy has been indiscriminately used as a very approximate measure of staff busyness or workload. Hence busyness is independent of unit size and should more accurately be measured using staff to patient ratios. Hence you need to point out those studies where % occupancy is masquerading as busyness with a set of negative outcomes specific to staff workload.

* Placing of patients into other wards has a sound theoretical basis in queuing theory and very much depends on the size of the bed pool. You are advised to find a study titled 'Hospital bed occupancy demystified' which links hospital average occupancy to hospital size and the size of the bed pools. The Erlang B equation shows the level of turn-away associated with the interplay between average occupancy and bed pool size. Erlang B depends on Poisson randomness in arrivals. Lines of constant turn-away can be generated and used as a means of creating a framework to compare units of different size. See a recent study 'Capacity planning for small hospitals and departments' for a wider discussion. More recent studies on the topic are available and the term 'turn-away' may be worth pursuing.

* On this point maybe re-read the study of Bain et al and see it in a different light. Also in the MJA was another study titled 'Myths of ideal hospital size' which is also relevant.

* The fact that the largest hospital in your study accounts for 50% of admissions therefore skews the whole meaning of % occupancy. Please provide descriptive data for each of the hospitals in the study such as total beds and the sizes of the relevant bed pools and average occupancy which I think can be estimated. May I suggest splitting your data between the large hospital and the other smaller hospitals to see if the associations with % occupancy change. This is a central weakness in the study since weighted population density (WPD) of world countries seems to dictate the size range of hospitals possible in each country, see the small hospitals and departments study. Many countries have WPD lower than Sweden and hence will operate at much lower average % occupancy. This explains why different countries have different average bed occupancies in their hospitals as per OECD data.

* It would be helpful if you could point out that bed occupancy shows a 24-hour cycle, see 'Understanding the current state of patient flow in a hospital' and 'Do Midnight Censuses Accurately Portray Hospital Bed Occupancy?'

* Hence you need to explain why 11 am was chosen as the base point for bed occupancy and if possible, provide an example of the 24-hour cycle. The key point then becomes the proportion of time that the beds are at 100% occupancy during the day or season of the year.

* Out of specialty bed placement for the medical specialties occurs most frequently in the winter months when circulating pathogens lead to a surge in respiratory admissions which then overflow to surrounding wards. Clearly this varies from winter to winter.

* This effect is related to the nearness to death or time to death effect where over 50% of a person's lifetime hospital bed occupancy occurs in the last year (mostly last 6 months) of life. This is irrespective of the age at death. Hence age means slightly different things in different countries depending on the most frequent age at which death occurs. The absolute number of deaths then becomes a lagging indicator of medical bed pressures and indeed of future bed requirements.

* Perhaps add a sentence explaining that socioeconomic status is quantified in different countries depending on available data and this will affect different studies on the topic. I suspect that education level is the most important.

* Bed occupancy >100%. This needs greater explanation. Years ago, in England there was a category called 'ward attenders' which were patients coming back to the ward to have dressings changed, etc. Most did not actually require a bed. You need to describe the circumstances surrounding this group of patients.

* In England there is a group of staff called 'bed managers' who manage the process of patients queuing for admission when beds are 100% occupied. Are bed managers involved in the decision in your study? Who makes the decision?

Other than the above comments the other parts of the introduction are excellent and very comprehensive.

Hence in summary, qualify what % occupancy implies and what different literature studies are implying but often not stating. Further explain the context of your study. Most importantly check if the statistical associations in the study differ between the large (tertiary?) hospital and the other smaller hospitals.

The study is an important contributor to this field but requires the necessary additional explanations to improve its far wider application to the many smaller hospitals in the world.

I look forward to seeing the revised version.

VERSION 1 - AUTHOR RESPONSE

Reviewer: 1
Dr. Evan Newnham, Eastern Health, Monash University
Comments to the Authors:
This is interesting subject matter not previously explored in this way.

1. Introduction: Is there evidence that the maturation of hospital in the home models of care has also reduced demand on inpatient capacity (may strengthen the introduction).

We are following the hospital-in-home models with great interest. In Sweden, these models of care are fairly new in Sweden and we have not found any studies that have evaluated the impact of hospital in home models on the need for hospital inpatient care. However, this may well be true in other countries and we have therefore clarified our opening statements about the shift from inpatient to outpatient care, see line 62.

2. The term 'bedspacing' is not in common usage in the antipodes - an early reference to the terminology would help the non-European reader.

There is indeed no consensus in the literature regarding the term that should be used to describe this process. We chose bedspacing as the term used in our paper mainly because other terms that are used for this process can easily be misinterpreted in scientific text. For instance, 'outliers' often refer to extreme observations rather than patients placed in off-service wards and the term ´boarding´ more often than not refers to patient kept in emergency departments. We have revised the initial sentences in the section about bedspacing so that it now includes common terminology for the process of bedspacing, see line 114.

3. The introduction (particularly the 'bedspacing' section goes into significant detail regarding patient outcomes related to bedspacing and could be substantially shortened.

We partly agree with the reviewer that the section ´bedspacing´ can be shortened. However, we also believe that it is important to elaborate on the gaps in the scientific literature in order to justify why this study is needed. We have revised the section 'bedspacing' and shortened it to the extent we believe is possible without losing important context regarding the current scientific knowledge.

4. Note the inconsistency in dates in the 'study population section' - please address.

We thank the reviewer for noticing this, the format and wording have been revised, see line 224.

5. The term 'sex' is used a few times and could be replaced with 'gender'.

We thank the reviewer for this comment as it is an important subject in relation to register based studies. The variable sex corresponds to the legally registered sex and in these registers, the vast majority of patients have been registered based on their biological traits at birth (sex). However, some patients may have changed their registered sex in order to align with their social identity. However, the data does not capture gender per se. As such, we have changed the term sex to registered sex throughout the manuscript.

6. A substantial components of the results discuss the clinical outcomes of the patient cohorts - noting the primary purpose of the paper is concentrated on demographic differences, the results section could also be shortened.

The reviewer is correct that the primary purpose of the paper is to investigate the relationship between bed occupancy, patient charachteristics and bedspacing. The two clinical outcomes reported in the results is the need for intensive care and complications of care. Given the data we

have, we do believe that these two outcomes are important to mention in the results as they are primary concerns regarding bedspaced patients. However, we have noticed that the clinical outcomes are reported twice in the results section, we have therefore simplified this by only mentioning the clinical outcomes in one paragraph, see the 'Results' section line 361.

7. As a general comment, the paper could be shortened to focus more on the stated main objectives.

We agree with the reviewer and have shortened the text wherever it was appropriate to do so.

Reviewer: 2
Dr. Rodney Jones, Healthcare Analysis & Forecasting
Comments to the Authors:
This is a valuable study which identifies the conditions, ages and socioeconomic factors associated with patients placed outside their most suitable specialty.

We thank the reviewer for this supportive comment.

May I suggest some improvements to enhance the study:

1. I had not come across the term bed spacing before. In the USA this is called off-service placement and in the UK called bed boarding or outliers It may be useful to see if any other terms are used and inform the readers accordingly. Perhaps point out that the terminology varies around the world. In the UK the term boarding can be used in both an ED and inpatient context. Also, in the UK the term outlyer is also used in an inpatient context. You may have missed relevant UK studies due to terminology.

We are aware that there is different terminology used depending on the context and have used terms such as boarding and outliers in our literature searches. As we have an ambition to further investigate bedspacing – including the effects on patient safety – we have decided to use the terms used by other authors who have made great contributions to the field, for instance the study "*Bedspacing and clinical outcomes in general internal medicine"* by Zannella et al. However, the reviewer is correct in pointing out that these are not mentioned in the background. As such, we have added a sentence describing the different terminology in the first part of the section called 'Bedspacing', see line 114.

2. In the introduction you need to mention that average bed occupancy has also been increasing over time indicating that bed supply is declining faster than the savings from technological change.

We believe that this is indeed true. However, data regarding bed occupancy has been difficult to obtain in Sweden and we have no direct evidence of this in our local context. We have however revised our text so that it now reflects on the mismatch between the decline in supply and possible savings from medical advancements see line 75.

3. You probably need to mention the nearness to death or time to death effect where 55% of a person's lifetime bed occupancy occurs in the last year of life irrespective of the age at death. In this case the trend in the absolute number of deaths becomes a predictive variable in bed demand.

We thank the reviewer for pointing this out and have added a few sentences in the introduction where we reflect on the increasingly older population and the expected increase in demand for hospital services as more patients in total will be in their last year of life, see line 67.

The idea of predicting bed demand based on the absolute number of deaths is very interesting. We briefly looked into this and the annual total mortality in Sweden has been ranging from 90 000 to 95 000 during the last 20 years, despite a substantial population increase (over 15%) during the same time period. At the same time, anecdotal evidence tells us that bed occupancy has increased substantially in most Swedish hospitals, although this is difficult to prove as the data is not available. As such, there seems to be other factors at play in our setting. Regardless, we thank the reviewer for this idea and we will keep it in mind in our future studies.

4. The issue of hospital bed occupancy needs further elaboration. One study titled 'Hospital bed occupancy demystified' may give you some leads. More recent studies on the topic are available and the term 'turn-away' may be worth pursuing.

We read, with great interest, the study 'Hospital bed occupancy demystified' and we thank the reviewer for pointing us to a paper which introduces queuing theory and erlang B models, something we have not studied in-depth previously. We have elaborated on the consequences of high bed occupancy and introduced the term 'turn-away' in the introduction, see the section 'Bedspacing'. Second, we have elaborated on what determines the bed occupancy threshold for patient safety, including hospital size, type of clinical specialty and whether care is planned or unplanned, see line 91.

5. Turn-away and bed occupancy are interlinked and depend on the size of the hospital, hence, you need to give some detail regarding the size of the hospitals in the study and check if the off-service placement is affected by hospital size.

We thank the reviewer for pointing out the potential differences between small and large hospitals, this is definitely a factor that we have overlooked as we have been focusing on hospital departments.

In the studied health care region, patients are cohorted into clinical departments where the departments consist of one or several wards for inpatient care together with ambulatory care facilities within the same clinical specialty. These clinical departments form the basis of our study with patients being placed outside of these departments categorized as bedspaced (exceptions from this rule can be seen in the methods section). The reasoning was that different departments – seeing different types of patients – may be more or less inclined to bedspace their patients depending on the difficulty of the needed treatment. As such, we chose to include models with random intercepts for the departments.

However, the reviewer makes an excellent point that hospital size may be of importance as well. Although speculative, we believe that although bed occupancy may differ between hospitals, the more important issue may be the availability of similar departments that can receive bedspaced patients without a negative impact on patient safety. For instance, in larger hospitals, it might be possible to bedspace patients to departments with a relatively similar clinical specialty. In smaller hospitals, this might not be possible to the same extent, with bedspacing more likely to occur between for instance a medical and a surgical department, i.e., bedspacing that is considered more dangerous in practice.
Although the impact of hospital size on bedspacing is not a stated aim in our study, we have:

a) added descriptive information about the hospitals under the heading ´Setting´ in the methods section and in a new table 1, see line 672.

b) Performed a post-hoc analysis to explore the relationship between bed occupancy and bedspacing separating large and small hospitals, see supplementary figure 4 and supplementary table 3.
c) Updated results and discussion to reflect the post-hoc analysis.

6. Indeed the 85% occupancy rule has no proof and has been shown to be entirely inappropriate for small units. In the literature 85% occupancy is incorrectly used as a proxy for staff busyness. Busyness generates a range of adverse outcomes but does not generate turn-away per se.

We agree with the reviewer that the 85% bed occupancy is not supported by scientific evidence. However, the 85% occupancy rule is often used in practice. For instance, the Swedish Government Agency responsible for health care (National Board of Health and Welfare) has repeatedly published guidelines recommending hospitals to aim for less than 85% occupancy in acute care and 95% occupancy for planned hospital care (reference [Target values for bed occupancy and staffed beds]). As such, we believe that using lower than 85% occupancy rate as a baseline is relevant in our case.

However, we also acknowledge that occupancy is a crude measurement. In a simple example, transferring "medically fit" patients to an off-service ward (bedspacing) while admitting the same number of more frail patients may very well increase nurse workload without affecting occupancy.

However, and as alluded to in our paper and in your next comment, Sweden has the lowest level of available beds in the developed world with inpatient hospital care to some extent reserved for the "most ill among the ill". As such, occupancy rates could to some extent be assumed to be highly correlated with nurse workload. We have elaborated on this in the section 'Management of hospital beds' (line 91), 'Bedspacing' (line 159) and 'Discussion' (line 482).

Other than the above comments the other parts of the introduction are excellent and very comprehensive.

We thank the reviewer for the encouraging comment.

7. Regarding beds in Sweden it has been confirmed that Sweden has the lowest level of available beds per death in the developed world, as per the nearness to death argument above. In this study deaths per 1000 population was used as a measure of population age profile from a nearness to death perspective, i.e., age per se is an unreliable indicator.

We are not surprised that Sweden has the lowest level of available beds per death in the developed world. What is often not mentioned in discussions regarding hospital capacity in Sweden is the extensive use of medicalized nursing homes in the last years of life. As such, for individuals in their last years of life, length of stays in hospitals are rather short as patients are discharged to these nursing homes as soon as possible.

8. Socioeconomic status needs to be qualified as different measures can be used in different countries.

We thank the reviewer for bringing this up, we have elaborated on how the socioeconomic variables were measured and added references for the interested reader.

** **

Further comments:

9. The most important issue lies around the theoretical basis for this behaviour. The figure of 85% has no theoretical basis and absolutely does not apply to small departments such as maternity, paediatrics or critical care. Strictly speaking it only approximately applies to very large hospitals but not to the bed pools within these hospitals.

The reviewer makes an excellent point in that the 85% figure does not apply to certain departments such as maternity, paediatric or critical care. Please note that for this reason, patients admitted to maternity wards or paediatric wards were excluded from our study population, see line 224 Furthermore, although this was not stated in our original manuscript, patient in ICU did not contribute to the calculation of bed occupancy.

However, as we have stated earlier, the 85% recommendation is used in practice in Sweden and could perhaps be argued to be important as relevant baseline this particular context. This is to some extent supported by the results as there is a HR = 1.9 of bedspacing when comparing category 85-100% with less than 85% occupancy.

10. In the literature % occupancy has been indiscriminately used as a very approximate measure of staff busyness or workload. Hence busyness is independent of unit size and should more accurately be measured using staff to patient ratios. Hence you need to point out those studies where % occupancy is masquerading as busyness with a set of negative outcomes specific to staff workload.

We agree that the inclusion of a more direct measurement of staff to patient ratios would be desirable in this study. Unfortunately, such data is not available to us. However, measuring staff to patient rations introduces additional problems as the appropriate ratio of staff / patients likely differs depending on the type of hospital department. Thus, the appropriate ratio of staff / patients will depend on the types of patients usually cared for in a department which requires in depth clinical expertise in order to assess an appropriate threshold for safe bed occupancy for different departments.

The data used in our study relies on the operative assessment of daily capacity made by chief physicians at the different departments. As such, the measurements of staffed beds available to us may be a good approximation of true capacity. We have clarified this under the heading ´Bed occupancy´ in the section ´Variables´, see line 245.

11. Placing of patients into other wards has a sound theoretical basis in queuing theory and very much depends on the size of the bed pool. You are advised to find a study titled ‘Hospital bed occupancy demystified’ which links hospital average occupancy to hospital size and the size of the bed pools. The Erlang B equation shows the level of turn-away associated with the interplay between average occupancy and bed pool size. Erlang B depends on Poisson randomness in arrivals. Lines of constant turn-away can be generated and used as a means of creating a framework to compare units of different size. See a recent study ‘Capacity planning forrn small hospitals and departments’ for a wider discussion. More recent studies on the topic are available and the term 'turn-away' may be worth pursuing.

A summarized response for 11 and 12 can be seen under comment 12.

12. On this point maybe re-read the study of Bain et al and see it in a different light. Also, in the MJA was another study titled 'Myths of ideal hospital size' which is also relevant.

We acknowledge that our perspective, which have been focusing on patient safety, could be improved by also including reasoning and knowledge from the field of capacity planning and queuing theory.

The issue of bed pools is particularly relevant as some bed pools may be sound from a queuing theory perspective – but not necessarily from a patient safety perspective. For instance, we have anecdotal evidence from a nearby hospital where bed pools span several different medical and surgical disciplines with obvious risks of delayed treatments and medical errors when contrasted to being treated and cared for by the most suitable discipline (effectively asking: do we need more staffed hospital beds?).

This is the reason for why we, in our study, focus on the departments as the most clinically relevant bed pool. However, as alluded to in comment 4, we much appreciate the literature recommendations by the reviewer and we will definitely bring these perspectives into our future work that will hopefully investigate the appropriateness of bed pool silos versus bed pools with shared disciplines in relation to patient safety.

13. The fact that the largest hospital in your study accounts for 50% of admissions therefore skews the whole meaning of % occupancy. Please provide descriptive data for each of the hospitals in the study such as total beds and the sizes of the relevant bed pools and average occupancy which I think can be estimated.

We agree with the reviewer that our initial manuscript had overlooked the issue of hospital size. As seen in the response to comment 5, we revised our manuscript to include exploratory analyses regarding the impact of hospital size on bedspacing at different levels of bed occupancy. However, in this study we consider a patient to be bedspaced if he or she is placed in a ward at another department that belongs to a different discipline. As such, what is most important in the case of bedspacing is the bed occupancy at a particular department and thus, we argue that the relevant bed pool is in fact the department (often consisting of several wards). The departments, and thus what we consider to be bed pools, are not necessarily different in size between the university hospital and the other hospitals. It is however true that the departments differ in size and that this may impact the risk of being bedspaced. This is the reason for my we include random intercepts at the department level in our main analysis.

Nevertheless, we have revised table 1 so that it now includes:

- The number of departments per hospital
- Number of patient days per hospital as a proxy for total beds per hospital (total beds have changed during the study period in most hospitals)
- Average bed occupancy during the study period for each hospital (at 11 am)

May I suggest splitting your data between the large hospital and the other smaller hospitals to see if the associations with % occupancy change.

Here we refer back to our response to comment 5, we have performed a post-hoc analysis that can be seen in the supplementary file.

- This is a central weakness in the study since weighted population density (WPD) of world countries seems to dictate the size range of hospitals possible in each country, see the small hospitals and departments study. Many countries have WPD lower than Sweden and hence will operate at much lower average % occupancy. This explains why different countries have different average bed occupancies in their hospitals as per OECD data.

We have acknowledged this limitation in the discussion where we now reason about to what extent our results can be generalized when it comes to the risk of being bedspaced at different levels of bed occupancy, see line 494.

14. It would be helpful if you could point out that bed occupancy shows a 24-hour cycle, see 'Understanding the current state of patient flow in a hospital' and 'Do Midnight Censuses Accurately Portray Hospital Bed Occupancy?'. Hence you need to explain why 11 am was chosen as the base point for bed occupancy and if possible, provide an example of the 24-hour cycle. The key point then becomes the proportion of time that the beds are at 100% occupancy during the day or season of the year.

We agree with the reviewer that this needs to be clarified further. First of all, we have included a visualization of the 24-hour cycle using data from the health care region studied, see supplementary figure 1. This figure largely confirms the observations from the study "Do midnight Census Accurately Portray Hospital Bed occupancy" by Riahi et al with a peak in bed occupancy at 10-11 am.

Furthermore, we believe that 11 am is an appropriate time to measure bed occupancy as it coincides with the peak occupancy. In practical terms this means that the staff is both planning and treating the newly admitted patients as well as preparing and planning for discharging patients in the afternoon. As such, we believe that bed occupancy at 11am is appropriate as it 1) generally coincides with the busiest period of the day and 2) is the time where clinical staff can assess the need for bedspacing depending on the inflow versus the expected outflow of patients. We have elaborated on this under the heading ´Bed occupancy´ in the variables section, see line 249.

15. Out of specialty bed placement for the medical specialties occurs most frequently in the winter months when circulating pathogens lead to a surge in respiratory admissions which then overflow to surrounding wards. Clearly this varies from winter to winter. This effect is related to the nearness to death or time to death effect where over 50% of a person's lifetime hospital bed occupancy occurs in the last year (mostly last 6 months) of life. This is irrespective of the age at death. Hence age means slightly different things in different countries depending on the most frequent age at which death occurs. The absolute number of deaths then becomes a lagging indicator of medical bed pressures and indeed of future bed requirements.

We thank the reviewer for the insight given on this matter. In our study we did not include seasonal adjustment directly as we are mainly interested in the relationship between bed occupancy and bedspacing, not whether this occurs in winter or not. However, the season might indeed impact the types of patients presenting to the hospital. As patients with certain diseases are more likely to be bedspaced, we therefore included adjustments of ICD-codes in our main analysis. The example of patients with infectious disease is particularly interesting as the possibility of bedspacing is not only dependent on whether there is a bed available elsewhere, but also if the bed is a single room bed or not. In addition to infectious disease, some hospitals in Sweden also experience surges of patients with fall-related injuries during late autumn/winter as snowfall starts.

16. Perhaps add a sentence explaining that socioeconomic status is quantified in different countries

depending on available data and this will affect different studies on the topic. I suspect that education level is the most important.

We have added a sentence about differences in quantifying socioeconomic status in the discussion, see line 425.

17. Bed occupancy >100%. This needs greater explanation. Years ago, in England there was a category called 'ward attenders' which were patients coming back to the ward to have dressings changed, etc. Most did not actually require a bed. You need to describe the circumstances surrounding this group of patients.

We thank the reviewer for pointing this out. In the at the included hospitals (and most hospitals in Sweden) in- and outpatient care is separated. A patient who has undergone surgery and needs to have dressings changed after being discharged will either go to the surgical department's outpatient clinic (separated staff) or receive help by primary care or home care services. The exception is if there is a complication that is so severe that the patient has to be readmitted. As such, the 'ward attenders´ are not contributing to inpatient bed occupancy. We have changed the Section "Study design and setting" to two separate sections where we elaborate on this.

18. In England there is a group of staff called 'bed managers' who manage the process of patients queuing for admission when beds are 100% occupied. Are bed managers involved in the decision in your study? Who makes the decision?

We thank the reviewer for bringing this into attention, it was a miss on our end. In brief, the decision to admit and bedspace is taken by physicians at the ED and relevant department, the task to find a bed is often given to a bed manger. We have clarified this under the section 'Setting'.

Hence in summary, qualify what % occupancy implies and what different literature studies are implying but often not stating. Further explain the context of your study. Most importantly check if the statistical associations in the study differ between the large (tertiary?) hospital and the other smaller hospitals.

We thank the reviewer for the comments and we hope that we have addressed these issues sufficiently.

The study is an important contributor to this field but requires the necessary additional explanations to improve its far wider application to the many smaller hospitals in the world.

I look forward to seeing the revised version.

VERSION 2 - REVIEW

**Reviewer**	**1**
**Name**	**Newnham, Evan**
**Affiliation**	**Eastern Health**

**Date**	**13-Oct-2026**
**COI**	

Thank you for considering the feedback in the revised document - this reads well.

**Reviewer**	**2**
**Name**	**Jones, Rodney**
**Affiliation**	**Healthcare Analysis & Forecasting**
**Date**	**22-Sep-2026**
**COI**	

Your comments to both reviewers were very poistive. In your #6 you noted that governments around the world have blindly recommended below 85% average occupancy without giving further context. The OECD and other have fallen into the same trap when publishing materials relevant to the topic. Alas the literature is full of studies with hidden assumptions and people citing 85% occupancy somewhat mindlessly. Congratulations on an exceptionally useful study and sucess for your further studies.
