# Supplementary material for: Influence of bed occupancy and patient characteristics on bedspacing practices: a multicentre retrospective cohort study
Source: BMJ Open. 2026 Oct 26;16(10):e120864. doi: 10.1136/bmjopen-2026-120864 (PMC13711533; doi:10.1136/bmjopen-2026-120864)
Supplement: Supplementary file 2 [file bmjopen-16-10-s001.docx]

# Supplementary figures

## Supplementary figure 1. 24-hour cycle of bed occupancy

Broken line graph showing the average bed occupancy per hour of day based on hourly averages from all hospital wards included in the analysed dataset.

## Supplementary figure 2. Average bed occupancy.

Histogram of bed occupancy based on daily observations at 11:AM from all departments included in the analysed dataset. Dashed lines represent the cut-offs used in the analysis.

## Supplementary figure 3. Average bed occupancy by hospital size

Histogram of bed occupancy based on daily observations at 11:AM from all departments included in the analysed dataset. Left graph represents the large university hospital and the right graph represents the regional general hospitals. Dashed lines represent the cut-offs used in the analysis.

## Supplementary figure 4. Predicted probabilities of bedspacing by hospital size

Predicted probabilities of bedspacing in relation to bed occupancy for large and small hospitals based on a univariate generalised linear model using natural splines.

## Supplementary figure 5. Pair of clinics involved in bedspacing in the sub-analysis.

Alluvial plot showing the 15 most common pairs of clinics that are involved in bedspacing in the sub-analysis. The left columns represent the cohorting clinic that are medically responsible for the patient whereas the right column represents the off-service clinic where the patient is physically present. The thickness of the ribbons represents the relative number of patients who were bedspaced in the particular pair of departments.

# Supplementary tables

## Supplementary table 1. Operationalisation of bedspacing

Table showing the operationalisation of bedspacing in the primary and secondary research objectives.

| **Primary treatment modality** | **Cohorting** | **Off-service** | **Bedspacing in the primary research objective** | **Bedspacing in the secondary research objective** |
| --- | --- | --- | --- | --- |
| Medicine | General internal medicine | Cardiology | Yes | No |
| Medicine | General internal medicine | Surgery | Yes | Yes |
| Surgery | Surgery | Orthopedy | Yes | No |
| Surgery | Surgery | General internal medicine | Yes | Yes |

## Supplementary table 2. Data selection

Table showing exclusion criteria used when generating analysed dataset.

| **Description** | **Patient days remaining in dataset after exclusion of:** |
| --- | --- |
| Total | 4 426 363 |
|  |  |
| - Missing ward data^a^ | 4 328 084 |
| - Planned visits^b^ | 3 546 422 |
| - Maternity / palliative units | 3 535 048 |
| - Psychiatric spells | 3 521 619 |
| - External wards^c^ | 3 485 717 |
| - Specialised wards^d^ | 3 463 501 |
| - Restricting spells to days-to-event | 3 040 255 |
| ^a^ Episodes with missing data for the number of staffed beds  ^b^ Not admitted from emergency departments or not admitted (unplanned) from outpatient clinics  ^c^ Short-term nursing home beds  ^d^ Wards used for specialised treatment and diagnostics (e.g., radiation therapy) | |

## Supplementary table 3. Characteristics of patients in the included hospital spells

| **Characteristic** | **Overall spells N = 480,245^1^** | **Cohorted spells N = 444,667^1^** | **Spells with bedspacing  N = 35,578^1^** | **p-value^2^** |
| --- | --- | --- | --- | --- |
| Age (mean, SD) | 68  (19) | 68  (19) | 68  (21) | <0.001 |
| Female (%) | 243,770  (51%) | 224,191  (51%) | 19,579  (55%) | <0.001 |
| CCI (mean, SD) | 1.21  (1.66) | 1.22  (1.65) | 1.19  (1.72) | <0.001 |
| DRG (mean, SD) | 0.91  (0.94) | 0.91  (0.94) | 0.98  (0.90) | <0.001 |
| **Marital Status (%)** |  |  |  | <0.001 |
| divorced | 90,155  (19%) | 83,454  (19%) | 6,701  (19%) |  |
| married | 200,743  (42%) | 187,366  (42%) | 13,377  (38%) |  |
| unmarried | 94,646  (20%) | 87,206  (20%) | 7,440  (21%) |  |
| widow(er) | 93,739  (20%) | 85,756  (19%) | 7,983  (22%) |  |
| **Highest level of education completed (%)** |  |  |  | 0.065 |
| Primary | 166,659  (35%) | 154,355  (35%) | 12,304  (35%) |  |
| Secondary | 195,733  (41%) | 181,183  (41%) | 14,550  (41%) |  |
| Tertiary | 106,622  (22%) | 98,801  (22%) | 7,821  (22%) |  |
| Unknown | 10,269  (2.1%) | 9,443  (2.1%) | 826  (2.3%) |  |
| **Region of birth (%)** |  |  |  | <0.001 |
| Europe | 58,990  (12%) | 54,357  (12%) | 4,633  (13%) |  |
| Outside of Europe | 28,165  (5.9%) | 26,020  (5.9%) | 2,145  (6.0%) |  |
| Sweden | 393,035  (82%) | 364,238  (82%) | 28,797  (81%) |  |
| Days to event^3^ (mean, SD) | 6  (7) | 7  (8) | 2  (4) | <0.001 |
| Length of Stay^4^ (mean, SD) | 8  (8) | 8  (9) | 8  (7) | <0.001 |
| ED visits last 12 months (mean, SD) | 1.44  (2.98) | 1.44  (2.98) | 1.52  (3.06) | <0.001 |
| Care in ICU (%) | 30,463  (6.3%) | 29,878  (6.7%) | 585  (1.6%) | <0.001 |
| Complication of care during hospital visit (%) | 12,829  (2.7%) | 11,560  (2.6%) | 1,269  (3.6%) | <0.001 |
| Bedspaced at hospital admission | 26,310  (5.5%) | 0  (0%) | 26,310  (74%) | <0.001 |
| Bedspaced at hospital discharge^5^ | 5,753  (1.2%) | 0  (0%) | 5,753  (16%) | <0.001 |
| **ICD-chapters (top 10, descending order)** |  |  |  | <0.001 |
| Circulatory System | 93,620  (19%) | 90,033  (20%) | 3,587  (10%) |  |
| Symptoms not elsewhere classified | 61,521  (13%) | 57,364  (13%) | 4,157  (12%) |  |
| Digestive System | 54,010  (11%) | 47,649  (11%) | 6,361  (18%) |  |
| Respiratory System | 50,825  (11%) | 46,815  (11%) | 4,010  (11%) |  |
| Injury, poisoning and other external causes | 55,135  (11%) | 49,961  (11%) | 5,174  (15%) |  |
| Genitourinary System | 31,150  (6.5%) | 28,289  (6.4%) | 2,861  (8.0%) |  |
| Infectious diseases | 22,179  (4.6%) | 20,425  (4.6%) | 1,754  (4.9%) |  |
| Neoplasms | 23,110  (4.8%) | 21,216  (4.8%) | 1,894  (5.3%) |  |
| Musculoskeletal System | 16,689  (3.5%) | 14,967  (3.4%) | 1,722  (4.8%) |  |
| Nervous System | 15,252  (3.2%) | 14,607  (3.3%) | 645  (1.8%) |  |
| Abbreviations: SD = Standard Deviation CCI = Charlson Comorbidity Index, DRG = Disease Related Groups, ICU = Intensive Care Unit | | | | |
| ^1^Number of hospital spells | | | | |
| ^2^Wilcoxon rank sum test; Pearson's Chi-squared test | | | | |
| ^3^ Day to event was calculated the number of days from admission to event (death, discharge, bedspaced) | | | | |
| ^4^ Length of Stay was calculated as the number of days from first day of hospital spell up until being discharged from the hospital | | | | |
| ^5^ Bedspaced at hospital discharge indicates whether the patient was bedspaced in the final day of the hospital spell^.^ | | | | |

## Supplementary table 4. The effects of patient characteristics and bed occupancy on bedspacing

| **Variable** | **Model 1** | **Model 2** | **Model 3** | **Model 4** |
| --- | --- | --- | --- | --- |
| (Intercept) | 0.025 (0.02-0.03) | 0.029 (0.03-0.03) | 0.025 (0.02-0.03) | 0.018 (0.01-0.03) |
| **Bed occupancy (Ref: < 85%)** |  |  |  |  |
| 85 - 100% | 1.939 (1.87-2.01) | 1.811 (1.75-1.87) | 1.807 (1.75-1.87) | 1.036 (1-1.07) |
| 100 – 115% | 2.422 (2.34-2.51) | 2.165 (2.09-2.25) | 2.163 (2.08-2.24) | 1.187 (1.14-1.24) |
| >115% | 3.967 (3.79-4.15) | 3.357 (3.21-3.52) | 3.356 (3.2-3.51) | 1.46 (1.39-1.54) |
| Day of visit | 0.678 (0.67-0.68) | 0.682 (0.68-0.69) | 0.682 (0.68-0.69) | 0.700 (0.7-0.7) |
| **Registered sex (Ref: Female)** |  |  |  |  |
| Male |  | 0.828 (0.81-0.85) | 0.852 (0.83-0.87) | 0.841 (0.82-0.86) |
| Age (scaled) |  | 0.927 (0.92-0.94) | 0.927 (0.92-0.94) | 0.954 (0.94-0.97) |
| CCI (weighted) |  | 0.987 (0.98-0.99) | 0.988 (0.98-1) | 0.993 (0.99-1) |
| **ICD-chapters (Ref: Certain Infectious and parasitic diseases)** |  |  |  |  |
| Diseases of the blood and blood forming organs |  | 1.059 (0.96-1.17) | 1.055 (0.96-1.17) | 1.01 (0.91-1.12) |
| Circulatory System |  | 0.541 (0.51-0.57) | 0.542 (0.51-0.57) | 0.575 (0.54-0.61) |
| Digestive System |  | 1.437 (1.36-1.52) | 1.443 (1.37-1.52) | 0.768 (0.72-0.81) |
| Genitourinary System |  | 1.245 (1.17-1.32) | 1.244 (1.17-1.32) | 1.232 (1.16-1.31) |
| Musculoskeletal System |  | 1.327 (1.24-1.42) | 1.325 (1.24-1.42) | 1.365 (1.27-1.47) |
| Nervous System |  | 0.584 (0.53-0.64) | 0.589 (0.54-0.64) | 0.52 (0.47-0.57) |
| Respiratory System |  | 1.029 (0.97-1.09) | 1.026 (0.97-1.09) | 0.981 (0.93-1.04) |
| Endocrine and metabolic diseases |  | 0.829 (0.76-0.9) | 0.823 (0.76-0.9) | 0.687 (0.63-0.75) |
| Factors influencing health status |  | 0.699 (0.63-0.78) | 0.698 (0.63-0.78) | 0.678 (0.61-0.76) |
| Injury, poisoning and other external causes |  | 1.234 (1.17-1.3) | 1.221 (1.16-1.29) | 0.971 (0.91-1.03) |
| Dementia |  | 0.976 (0.59-1.62) | 0.96 (0.58-1.6) | 0.924 (0.56-1.54) |
| Mental, Behavioural and Neurodevelopmental disorders |  | 0.605 (0.54-0.68) | 0.595 (0.53-0.67) | 0.398 (0.35-0.45) |
| Neoplasms |  | 1.003 (0.94-1.07) | 1.01 (0.94-1.08) | 0.771 (0.72-0.83) |
| other |  | 1.825 (1.7-1.97) | 1.821 (1.69-1.96) | 1.356 (1.26-1.46) |
| Pregnancy, childbirth and puerperium |  | 0.436 (0.38-0.5) | 0.434 (0.37-0.5) | 1.373 (1.16-1.63) |
| Symptoms not elsewhere classified |  | 1.026 (0.97-1.09) | 1.028 (0.97-1.09) | 0.917 (0.87-0.97) |
| **Highest level of education (Ref: Primary)** |  |  |  |  |
| Secondary |  |  | 1.013 (0.99-1.04) | 0.993 (0.97-1.02) |
| Tertiary |  |  | 1.034 (1-1.06) | 0.966 (0.94-1.00) |
| Unknown |  |  | 1.016 (0.95-1.09) | 0.975 (0.91-1.05) |
| **Marital Status (Ref: Married)** |  |  |  |  |
| Divorced |  |  | 1.059 (1.03-1.09) | 1.024 (0.99-1.05) |
| Unmarried |  |  | 1.15 (1.11-1.19) | 1.089 (1.05-1.12) |
| Widow(er) |  |  | 1.213 (1.18-1.25) | 1.194 (1.16-1.23) |
| **Region of Birth (Ref: Sweden)** |  |  |  |  |
| Europe |  |  | 1.101 (1.07-1.14) | 1.037 (1.00-1.07) |
| Outside of Europe |  |  | 1.122 (1.07-1.18) | 1.041 (0.99-1.09) |
|  |  |  |  |  |
| Department (random intercept SD) |  |  |  | 1.693 |
| Number of patient days^a^ | 3040255 | 3034389 | 3034389 | 3034389 |
| Number of departments |  |  |  | 74^b^ |
| AIC | 347459.4 | 342128.9 | 341905 | 310766.2 |
| BIC | 347524.1 | 342452.1 | 342331.6 | 311205.7 |
| *Note:* M1 is a model of the association between bed occupancy and bedspacing. M2 includes adjustment for clinical patient characteristics with M3 also adjusting for variables related to socioeconomic status. M4 is a mixed effects model and includes a random intercept for department. Random effects are reported as standard deviations. The effects are reported as hazard ratios with 95% confidence intervals in parenthesis.  ^a^ICD-codes were not available in 0,2% of the observed patient days. These observations were excluded from models M2 – M4.  ^b^Some departments placed their patients on shared wards. Out of the 82 departments included, there were 74 distinct bed pools. Patients that were transferred between these departments while remaining in the same bed pool were not considered to be bedspaced.  Abbreviations: CCI = Charlson Comorbidity Index, ICD = International Classification of Diseases, SD = Standard Deviation, AIC = Akaike Information Criterion, BIC = Bayesian Information Criterion | | | | |

## Supplementary table 5. The effects of bed occupancy on bedspacing at larger and smaller hospitals.

| Variable | Large hospital* | Small hospitals* |
| --- | --- | --- |
| (Intercept) | 0.036 (0.02-0.07) | 0.058 (0.03-0.11) |
| **Bed occupancy (Ref: <85%)** |  |  |
| 85 – 99% | 1.079 (1.03-1.13) | 1.046 (0.98-1.12) |
| 100 - 115% | 1.29 (1.22-1.36) | 1.131 (1.06-1.21) |
| >115% | 1.314 (1.22-1.42) | 1.6 (1.48-1.73) |
| Day of visit | 0.629 (0.62-0.63) | 0.791 (0.79-0.8) |
| *Note*: Table show the result of M1 (as applied in the main analysis) but with the dataset split between the large and the smaller hospitals. | | |

## Supplementary table 6. Characteristics of patients in the included hospital spells

Table shows the characteristics of patients in the included hospital spells using the stricter operationalisation of bedsacing.

| Characteristic | Overall spells N = 480,245^1^ | Cohorted spells N = 461,306^1^ | Spells with bedspacing  N = 18,939^1^ | p-value^2^ |
| --- | --- | --- | --- | --- |
| Age | 68  (19) | 68  (19) | 69  (21) | <0.001 |
| Female (%) | 243,770 (51%) | 233,163 (51%) | 10,607 (56%) | <0.001 |
| CCI (mean, SD) | 1.21  (1.66) | 1.22  (1.66) | 1.21 (1.70) | 0.007 |
| Mean DRG (SD) | 0.91  (0.94) | 0.91  (0.94) | 1.00 (0.98) | <0.001 |
| Marital Status |  |  |  | <0.001 |
| divorced | 90,155 (19%) | 86,499 (19%) | 3,656 (19%) |  |
| married | 200,743 (42%) | 193,870 (42%) | 6,873 (36%) |  |
| unmarried | 94,646 (20%) | 90,741 (20%) | 3,905 (21%) |  |
| widow(er) | 93,739 (20%) | 89,277 (19%) | 4,462 (24%) |  |
| Highest level of education completed |  |  |  | <0.001 |
| Primary | 166,659 (35%) | 159,923 (35%) | 6,736 (36%) |  |
| Secondary | 195,733 (41%) | 187,943 (41%) | 7,790 (41%) |  |
| Tertiary | 106,622 (22%) | 102,701 (22%) | 3,921 (21%) |  |
| Unknown | 10,269 (2.1%) | 9,820 (2.1%) | 449 (2.4%) |  |
| Region of birth |  |  |  | 0.003 |
| Europe | 58,990 (12%) | 56,542 (12%) | 2,448 (13%) |  |
| Outside of Europe | 28,165 (5.9%) | 27,125 (5.9%) | 1,040 (5.5%) |  |
| Sweden | 393,035 (82%) | 377,585 (82%) | 15,450 (82%) |  |
| Days to event^3^ (mean, SD) | 6  (7) | 6  (8) | 2  (4) |  |
| Length of Stay^4^ (mean, SD) | 8  (8) | 8  (8) | 7  (7) | <0.001 |
| ED visits last 12 months (mean, SD) | 1.44  (2.98) | 1.44  (2.98) | 1.57 (3.04) | <0.001 |
| Care in ICU (%) | 30,463 (6.3%) | 30,111 (6.5%) | 352 (1.9%) | <0.001 |
| Complication of care during hospital visit (%) | 12,829 (2.7%) | 12,226 (2.7%) | 603 (3.2%) | <0.001 |
| Bedspaced at hospital admission | 13,774 (2.9%) | 0 (0%) | 13,774 (73%) | <0.001 |
| Bedspaced at hospital discharge^5^ | 3,039 (0,6%) | 0 (0% | 3,039 (16%) | <0.001 |
| **ICD-chapters (top 10)** |  |  |  | <0.001 |
| Infectious diseases | 22,179 (4.6%) | 21,172 (4.6%) | 1,007 (5.3%) |  |
| Circulatory System | 93,620 (19%) | 91,677 (20%) | 1,943 (10%) |  |
| Digestive System | 54,010 (11%) | 50,913 (11%) | 3,097 (16%) |  |
| Genitourinary System | 31,150 (6.5%) | 29,333 (6.4%) | 1,817 (9.6%) |  |
| Musculoskeletal System | 16,689 (3.5%) | 15,714 (3.4%) | 975 (5.1%) |  |
| Nervous System | 15,252 (3.2%) | 14,963 (3.2%) | 289 (1.5%) |  |
| Respiratory System | 50,825 (11%) | 48,611 (11%) | 2,214 (12%) |  |
| Injury, poisoning and other external causes | 55,135 (11%) | 52,332 (11%) | 2,803 (15%) |  |
| Neoplasms | 23,110 (4.8%) | 22,188 (4.8%) | 922 (4.9%) |  |
| Symptoms not elsewhere classified | 61,521 (13%) | 59,346 (13%) | 2,175 (11%) |  |
| Abbreviations: SD = Standard Deviation CCI = Charlson Comorbidity Index, DRG = Disease Related Groups, ICU = Intensive Care Unit | | | | |
| ^1^Number of hospital spells | | | | |
| ^2^Wilcoxon rank sum test; Pearson's Chi-squared test | | | | |
| ^3^ Day to event was calculated the number of days from admission to event (death, discharge, bedspaced) | | | | |
| ^4^ Length of Stay was calculated as the number of days from first day of hospital spell up until being discharged from the hospital | | | | |
| ^5^ Bedspaced at hospital discharge indicates whether the patient was bedspaced in the final day of the hospital spell^.^ | | | | |

## Supplementary table 7. The effects of patient characteristics and bed occupancy on bedspacing using the stricter operationalisation of bedspacing.

| Variable | Model 1 | Model 2 | Model 3 | Model 4 |
| --- | --- | --- | --- | --- |
| (Intercept) | 0.008 (0.01-0.01) | 0.01 (0.01-0.01) | 0.009 (0.01-0.01) | 0.001 (0-0) |
| **Bed occupancy (Ref: < 85%)** |  |  |  |  |
| 85 - 100% | 3.038 (2.87-3.21) | 2.796 (2.64-2.96) | 2.789 (2.63-2.95) | 1.259 (1.19-1.34) |
| 100 – 115% | 4.135 (3.9-4.38) | 3.715 (3.5-3.94) | 3.711 (3.5-3.94) | 1.443 (1.35-1.54) |
| > 115% | 6.233 (5.82-6.68) | 5.353 (4.99-5.74) | 5.353 (4.99-5.74) | 1.834 (1.7-1.98) |
| Day of visit | 0.692 (0.69-0.7) | 0.696 (0.69-0.7) | 0.695 (0.69-0.7) | 0.706 (0.7-0.71) |
| **Sex (Ref: Female)** |  |  |  |  |
| Male |  | 0.786 (0.76-0.81) | 0.813 (0.79-0.84) | 0.795 (0.77-0.82) |
| Age (scaled) |  | 0.937 (0.92-0.95) | 0.93 (0.91-0.95) | 0.896 (0.88-0.91) |
| CCI (weighted) |  | 0.99 (0.98-1) | 0.991 (0.98-1) | 1.014 (1-1.02) |
| **ICD-chapters (Ref: Certain Infectious and parasitic diseases)** |  |  |  |  |
| Diseases of the blood and blood forming organs |  | 1.133 (1-1.29) | 1.131 (1-1.28) | 1.08 (0.95-1.23) |
| Circulatory System |  | 0.525 (0.49-0.57) | 0.525 (0.49-0.57) | 0.583 (0.54-0.63) |
| Digestive System |  | 1.186 (1.1-1.27) | 1.193 (1.11-1.28) | 0.879 (0.81-0.95) |
| Genitourinary System |  | 1.401 (1.3-1.51) | 1.401 (1.3-1.51) | 1.329 (1.23-1.44) |
| Musculoskeletal System |  | 1.308 (1.2-1.43) | 1.308 (1.2-1.43) | 1.497 (1.36-1.64) |
| Nervous System |  | 0.457 (0.4-0.52) | 0.461 (0.4-0.53) | 0.457 (0.4-0.52) |
| Respiratory System |  | 0.993 (0.92-1.07) | 0.987 (0.92-1.06) | 0.994 (0.92-1.07) |
| Endocrine and metabolic diseases |  | 0.734 (0.65-0.83) | 0.726 (0.64-0.82) | 0.766 (0.68-0.86) |
| Factors influencing health status |  | 0.618 (0.53-0.72) | 0.619 (0.53-0.72) | 0.69 (0.59-0.8) |
| Injury, poisoning and other external causes |  | 1.164 (1.08-1.25) | 1.149 (1.07-1.24) | 1.16 (1.07-1.26) |
| Dementia |  | 1.229 (0.68-2.23) | 1.217 (0.67-2.21) | 1.055 (0.58-1.91) |
| Mental Behavioural Neurodevelopmental disorders |  | 0.68 (0.58-0.79) | 0.661 (0.57-0.77) | 0.548 (0.47-0.64) |
| Neoplasms |  | 0.835 (0.76-0.92) | 0.844 (0.77-0.93) | 0.753 (0.68-0.83) |
| other |  | 1.503 (1.36-1.67) | 1.497 (1.35-1.66) | 1.497 (1.35-1.66) |
| Pregnancy childbirth and the puerperium |  | 0.226 (0.17-0.31) | 0.229 (0.17-0.31) | 1.037 (0.74-1.45) |
| Symptoms not elsewhere classified |  | 0.95 (0.88-1.02) | 0.952 (0.88-1.03) | 0.949 (0.88-1.02) |
| **Highest level of education (Ref: Primary)** |  |  |  |  |
| Secondary |  |  | 1.004 (0.97-1.04) | 0.989 (0.96-1.02) |
| Tertiary |  |  | 0.973 (0.93-1.01) | 0.952 (0.91-0.99) |
| Unknown |  |  | 1.039 (0.94-1.15) | 0.971 (0.88-1.07) |
| **Marital Status (Ref: Married)** |  |  |  |  |
| Divorced |  |  | 1.105 (1.06-1.15) | 1.042 (1-1.09) |
| Unmarried |  |  | 1.186 (1.13-1.24) | 1.102 (1.05-1.15) |
| Widow(er) |  |  | 1.252 (1.2-1.31) | 1.212 (1.16-1.26) |
| **Region of Birth (Ref: Sweden)** |  |  |  |  |
| Europe |  |  | 1.077 (1.03-1.12) | 0.993 (0.95-1.04) |
| Outside of Europe |  |  | 1.046 (0.98-1.12) | 0.976 (0.91-1.04) |
|  |  |  |  |  |
| Department (random intercept SD) |  |  |  | 3.37 |
| Number of patient days^a^ | 3040255 | 3034389 | 3034389 | 3034389 |
| Number of departments^b^ |  |  |  | 74 |
| AIC | 208273.8 | 205373.4 | 205225.1 | 187415 |
| BIC | 208338.4 | 205696.6 | 205651.6 | 187854.5 |
| *Note:* M1 is a univariate model of the association between bed occupancy and bedspacing. M2 includes adjustment for clinical patient characteristics with M3 also adjusting for variables related to socioeconomic status. M4 is a mixed effects model and includes a random intercept for department. Random effects are reported as standard deviations. The effects are reported as hazard ratios with 95% confidence intervals in parenthesis.  ^a^ICD-codes were not available in 0,2% of the observed patient days. These observations were excluded from models M2 – M4.  ^b^Some departments placed their patients on shared wards. Out of the 82 departments included, there were 74 distinct bed pools. Patients that were transferred between these departments while remaining in the same bed pool were not considered to be bedspaced  Abbreviations: CCI = Charlson Comorbidity Index, ICD = International Classification of Diseases, SD = Standard Deviation, AIC = Akaike Information Criterion, BIC = Bayesian Information Criterion | | | | |
